# A rare case of acute liver failure with intrahepatic cholestasis due to dengue hemorrhagic fever: CytoSorb® and plasma exchange aided in the recovery: case report

**DOI:** 10.1186/s12879-022-07933-y

**Published:** 2022-12-13

**Authors:** Arosha Minori Gunasekera, Udeshan Eranthaka, Dilshan Priyankara, Ranjith Kalupahana

**Affiliations:** National Hospital of Sri Lanka, Colombo, Sri Lanka

**Keywords:** Cholestasis, Acute liver failure, Dengue fever, Dengue haemorrhagic fever, Therapeutic plasma exchange, Cytosorb®

## Abstract

**Background:**

Dengue haemorrhagic fever is a severe form of acute dengue infection characterized by leakage of plasma through capillaries into body spaces resulting in circulatory insufficiency leading to shock. Despite varying degrees of liver involvement occurring in acute dengue infection, intrahepatic cholestasis is very rare in the literature with only two cases reported so far. We report a challenging case of a middle-aged woman with DHF complicated by acute liver failure, coagulopathy, acute renal failure and prolonged intrahepatic cholestasis. She was successfully managed in the intensive care unit with supportive therapy, Cytosorb® and therapeutic plasma exchange.

**Case presentation:**

A 54-year-old Sri Lankan obese woman with multiple comorbidities presented with fever, headache, vomiting and generalized malaise for 3 days and was diagnosed with dengue haemorrhagic fever. Despite the standard dengue management, she clinically deteriorated due to development of complications such as, acute liver injury, intrahepatic cholestasis and acute renal injury. Acute liver failure was evidenced by transaminitis, lactic acidosis, coagulopathy with pervaginal bleeding and severe encephalopathy necessitating elective intubation and mechanical ventilation. She was immediately transferred to intensive care facilities where she underwent supportive management for liver failure, continuous renal replacement therapy coupled with cytosorb and therapeutic plasma exchange with which she made a remarkable recovery.

**Conclusion:**

Acute liver failure with a prolonged phase of intrahepatic cholestasis is a very rare complication of acute dengue illness which is sparsely documented in medical literature so far. This patient was managed successfully with supportive therapy, aided by cytoSorb hemo-adsorption and therapeutic plasma exchange.

## Background

Dengue is considered to be the most common mosquito-borne viral disease in humans. Approximately 400 million cases occur annually in tropical and subtropical parts of the world, out of which nearly 500,000 cases end up in severe disease (dengue haemorrhagic fever/dengue shock syndrome) and it accounts for over 20,000 deaths annually worldwide [1]. It is becoming a major health problem both globally and locally. Dengue is endemic in over 125 countries [1] including Sri Lanka.

Dengue is a single stranded, enveloped RNA virus which belongs to flavivirus family. It has four distinct serotypes (DENV 1–4), transmitted by infected Aedes aegypti and Aedes albopictus mosquito species.

Dengue haemorrhagic fever (DHF) is a severe form of the disease characterized by leakage of plasma through capillaries into body spaces resulting in inadequate circulatory volume, leading into shock.

Liver involvement in dengue infection is frequently observed and sometimes can lead to acute liver failure with fatal outcomes [2]. Varying degrees of liver involvement occurs in acute dengue infection, ranging from asymptomatic elevation of transaminases to fulminant hepatic failure. But intrahepatic cholestasis in acute dengue infection is very rare in literature with only two cases reported in the world so far [3].

We report a challenging case of a middle-aged woman with DHF complicated by acute liver failure (ALF), coagulopathy, acute renal failure (ARF) and prolonged intrahepatic cholestasis who made a successful recovery. She was managed in the intensive care unit with supportive therapy, Cytosorb® and therapeutic plasma exchange (TPE).

## Case presentation

A 54-year-old obese Sri Lankan woman (BMI of 32) presented with fever, headache, vomiting and generalized malaise for 3 days. She has a background medical history of type 2 diabetes mellitus, hypertension and right-sided ischemic stroke. On admission she was febrile (38.7 °C), with stable hemodynamics. Rest of the systemic examination was unremarkable including abdominal examination. Initial investigations revealed white cell count (WBC) of 5.9 (× 10 ^3^/ μL), Platelet count of 179 (× 10 ^3^/ μL), Hemoglobin of 12.8 g/dl, AST 47 (U/L), ALT 37 (U/L) and bilirubin levels within normal range. Next day (on day 5 of the illness) there was a drastic drop in platelet and WBC counts from initial values to 55 (× 10 ^3^/ μL) and 3.69 (× 10 ^3^/ μL) respectively, with dengue IgM antibodies being positive, which was confirmative of acute dengue infection. The patient was managed according to the national dengue guidelines with meticulous monitoring of vital parameters and urine output while carefully balancing the fluid intake. Over the next few hours her clinical condition deteriorated with tachycardia (heart rate 100 bpm) and low urine output of < 0.5 ml/kg/hour though she was maintaining a stable blood pressure at 140/90 mmHg and capillary refilling time less than 2 s. Bed side ultrasound scan showed free fluid in peritoneal cavity which was suggestive of DHF. Clinical picture was further complicated by acute per-vaginal bleeding with a drop in haematocrit by 20%. She was transfused with 1 unit of red cell concentrate in addition to crystalloids. Despite all these measures, her condition further deteriorated and she became drowsy.

Blood gas analysis revealed a metabolic acidosis, with a pH of 7.27, HCO3– 17.7 mmol/l and lactate of 2.7 mmol/l. Subsequent clinical course was complicated by acute renal injury, acute disseminated coagulopathy (DIC) and acute liver failure. ALF was diagnosed due to altered liver biochemistry (Table 1), coagulopathy and encephalopathy. She was started on intravenous N-acetyl cysteine (NAC) infusion and transferred to Medical Intensive Care Unit (ICU) at this point.

**Table 1:** Results of laboratory investigations

Investigation	Day of the illness										Follow up visit (day)
	4	5	6	7	8	9	13	18	19	20	50
Hb (g/dl)	12.8	14.2	13	11	10.1	8.1	9.8	9.6	9.3	9.4	11.6
Hct (%)	40.3	42.8	40.4	39	30.6	30	35	36	38	39	41
WBC (× 10 ^3^/ μL)	5.9	3.69	3.4	4.3	11	12	8	10.3	8	9.5	7
Platelet (× 10 ^3^/ μL)	179	55	7	10	11	53	37	91	98	150	322
AST (U/L)	47	227	14,275	5046	3975	2417	311	67	48	81	44
ALT (U/L)	37	129	4419	2338	2390	1799	500	82	19	14	37
ALP (U/L)					168		131			122	
GGT (U/L)					238		153			87	
Total Bilirubin (mg/dl) Ref range 0.2–1.2	0.6	0.5	1.3	4.9	4.9	6.1	10.8	18.6	16.2	15.6	1.2
Direct Bilirubin (mg/dl) Ref range 0–0.5	0.3	0.3	0.8	2.1	3.2	5	8.3	14.2	12	10	0.59
PT/INR			1.99	2.8	2.89		1.41		1.16	1.02	1.1
pH		7.3	7.27	7.23	7.3	7.3	7.4	7.4	7.41	7.39	
Lactate (mmol/l)		2.7	7.3	8.2	3.5	2.6	2	1.5	1.1	0.9	
Creatinine (mg/dl)	1.1	1.2	3.4	4.8	1.8	2.3	2.1	4.1		2.8	1.53
Events		Critical phase started	ICU admission & CRRT	CRRT + Cytosorb	CRRT + Cytosorb			TPE 1st cycle	TPE 2nd cycle	Extubate	

On arrival in the ICU, she was encephalopathic with a Glasgow coma scale (GCS) of 12–14 and was having oliguric AKI. Blood gases revealed worsening metabolic acidosis with rising lactates (2.7–4.1 mmol/l). Inflammatory markers like CRP were normal. Blood and urine cultures were sterile, excluding sepsis.

During next few days hepatic encephalopathy worsened and she was electively intubated due to low GCS. At this point, her non-contrast CT (NCCT) brain was normal and EEG (Electroencephalogram) showed acute encephalopathy changes.

**Fig. 1:**
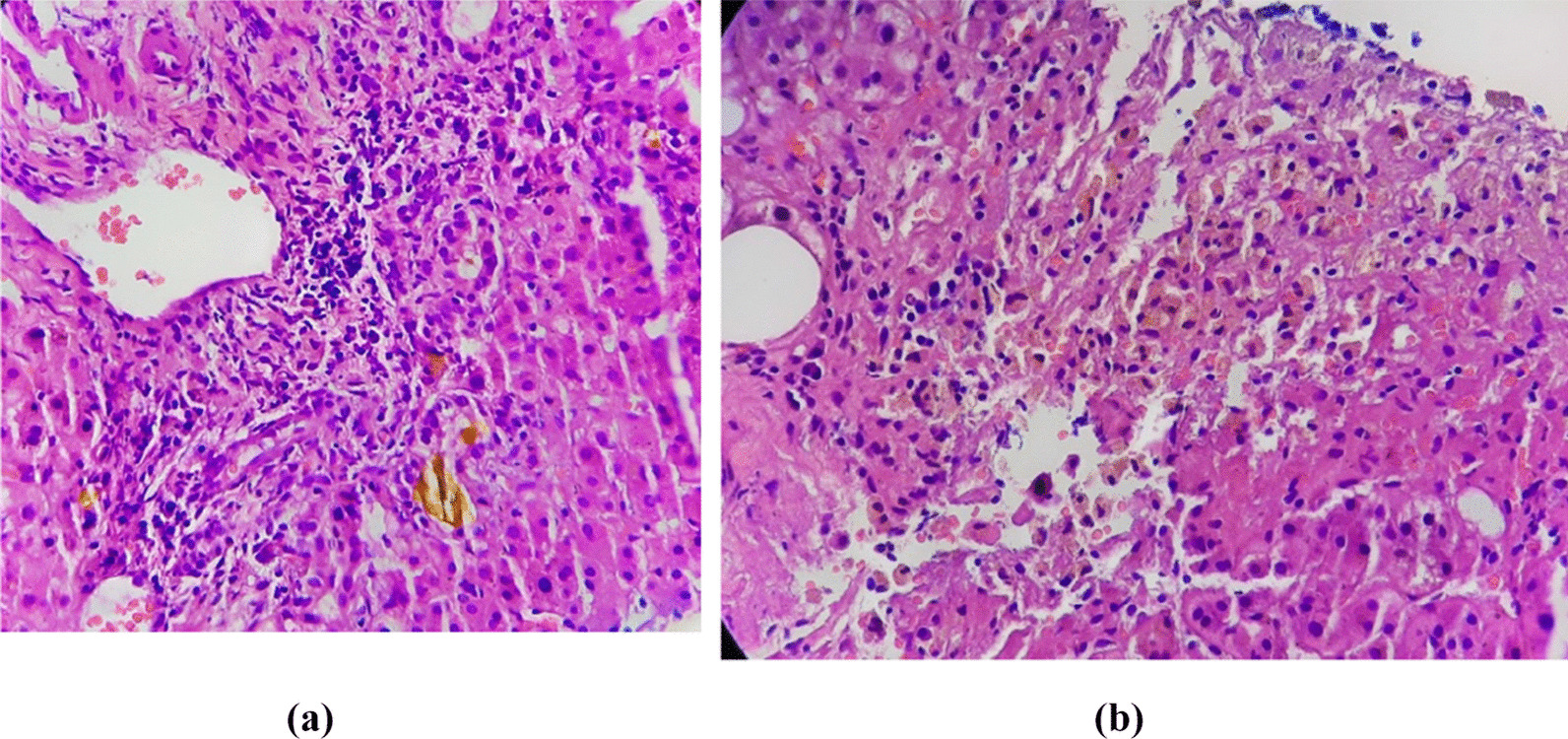
**A** and **B** Histology of the liver biopsy. (H&E stain × 400) The portal tracts are expanded by mixed inflammation with bile ductular proliferation, bile plugs and few neutrophils infiltrating the epithelium. The hepatocytes are bile stained; the sinusoids contain many Kupffer cells with spotty hepatocyte necrosis. The histological features are consistent with acute cholestatic hepatitis

Even though her transaminitis was settling, she had persistently rising direct bilirubinemia with high levels of alkaline phosphatase (ALP) and Gamma glutamyl transferase (GGT) (Table 1) with development of deep jaundice. On retrospective review, she had no exposure to drugs which can cause cholestasis or hepatotoxicity. CECT abdomen did not reveal extra-hepatic biliary obstructions. Hepatitis A, B, C, cytomegalovirus (CMV), Epstein Barr virus (EBV) and leptospira serology were negative as well as anti-nuclear antibody (ANA) and anti-mitochondrial antibody (AMA). Liver biopsy was performed after correction of coagulopathy and the histology revealed intra hepatic cholestasis (Fig. 1).

**Fig. 2:**
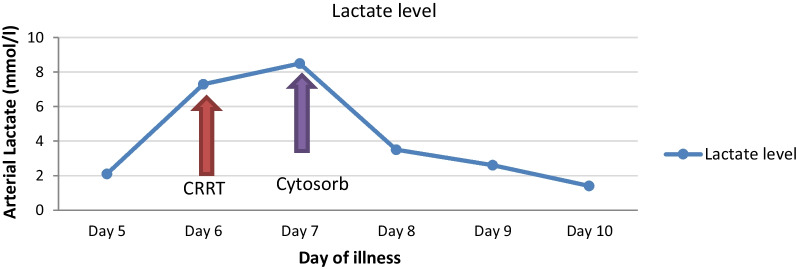
Arterial lactate behaviour during the illness. (Red arrow—the place where CRRT was started, purple arrow—the place where cytosorb was added)

She was initiated on continuous renal replacement therapy (CRRT) to stabilize metabolic parameters and to mitigate the worsening lactic acidosis. However, despite escalating dose of CRRT lactic acidosis got worsened (7.3–8.2 mmol/l). At this point CytoSorb® hemo-adsorption therapy was introduced coupled with CRRT after which metabolic parameters returned to normal with gradual drop in lactate with correction of acidosis (Fig. 2). She was transfused with fresh frozen plasma, platelet and cryoprecipitate guided by the ROTEM (rotational thromboelastometry) studies, following which her pervaginal bleeding settled and INR normalized.

She was started on therapeutic plasma exchange (TPE) due to persistently rising serum bilirubin levels and severe encephalopathy, after which the GCS started to improve and patient was safely extubated. 4 cycles of TPE was done with recovery of GCS to 15/15. Hyperbilirubinemia was treated with ursodeoxycholic acid as well. Patient was discharged home after good recovery.

On review 1 month later, she was asymptomatic, her bilirubin levels were reaching the baseline (Table 1) and repeat USS of liver was normal other than grade 2 fatty liver.

## Discussion and conclusion

Dengue infection, though asymptomatic in majority, has wide variety of clinical manifestations. It ranges from undifferentiated fever, dengue fever, DHF to expanded dengue syndrome [2]. Liver involvement is well-recognized in dengue infection, ranging from mild transaminitis to fulminant hepatic failure which has a high mortality [2]. A review of dengue deaths by Thulaseedharan and colleagues reported ALF as the most common cause for death in Dengue Fever [4]. Its true incidence and possible underlying risk factors for its development are poorly evaluated so far.

The pathophysiology of ALF in dengue is multifactorial and the mechanisms are still not well understood. Among the theories, three main mechanisms of dengue associated liver injury can be found such as, direct cellular apoptosis by dengue virus, ischemic hepatopathy and hypoxic injury due to poor hepatic perfusion following dengue shock and immune mediated end organ damage [2, 5, 8]. Dengue can replicate within hepatocytes causing a dysregulated host immune response [5]. Immune mediated injury is mainly mediated through cytokines which are produced in large amounts during acute infection to eliminate the virus. Furthermore, emerging evidence suggests that disturbances of liver microcirculation, and reduced portal blood flow which results in raised portal pressure and reversed portal blood flow could be contributing significantly to the pathogenesis of severe dengue related liver disease [6].

Generally, elevation of transaminases above thousand is accounted by acute ischemic hepatopathy, acute drug induced liver injury or acute viral hepatitis [9]. Since the patient had stable hemodynamics throughout the disease course, ischemic hepatic injury cannot be entertained in this case and drug induced liver injury was excluded as well.

In viral hepatitis other than dengue, ALT rise is usually higher than AST rise. However, reversed AST/ALT ratio is seen in dengue illness [5]. The exact cause for this reversal in dengue hepatitis is not well described. Unlike AST/ALT, serum bilirubin, ALP and G-GT do not show marked rise even in complicated dengue infections [10]. Moreover, there are only 2 reported cases of dengue fever with cholestatic liver disease in adults and one case of prolonged neonatal jaundice following dengue infection [3, 11]. The hypothesis for the cholestasis in these patients thought to be due to on-going inflammatory process of dengue infection.

Liver histological findings related to dengue infection are limited in literature since liver biopsy is not feasible in most cases of acute dengue infection due to thrombocytopenia and coagulopathy. Classic histological changes primarily involve the liver cells with sparing of the bile ducts [7]. In contrast the index case liver biopsy demonstrated an acute cholestatic hepatitis picture, which is an unusual manifestation in dengue.

In the index case, ALF was marked by features of severe liver injury such as very high transaminitis (AST > ALT) of more than thousands, coagulopathy with high PT/INR, encephalopathy and significantly raised lactate level despite good peripheral perfusion. Transaminases have peaked on day 6 of the illness and then subsided gradually. Even though transaminitis settled, there was a prolonged phase of direct hyperbilirubinemia with associated low GCS. Liver biopsy was performed at this stage which revealed acute cholestatic hepatitis (Fig. 1a, b).

Currently, there are no specific antiviral drugs aimed at treating dengue illness despite its growing incidence. As a result of limited therapeutic strategies and limited studies for novel treatment options, management of dengue and dengue associated liver failure is mainly supportive with meticulous fluid management, correction of hypoglycemia, coagulopathy and electrolyte imbalance.

Despite the scarcity of evidence, we have used cytosorb and TPE in the management of this patient with a successful outcome. Cytosorb® is a polymer filter, which can adsorb higher molecular weight components in plasma. It was mainly studied in the setting of sepsis and septic shock as a cytokine remover. We used Cytosorb in conjunction with continuous renal replacement therapy (CRRT) as the patients’ clinical condition deteriorated with worsening lactic acidosis. We observed a dramatic reduction in lactate levels with the use of Cytosorb®

There have been only a few case reports in the literature where Cytosorb® has been used in DHF [12, 13]. In addition to removing various endogenous and exogenous compounds such as myoglobin and bilirubin, Cytosorb® therapy has shown to modulate the immune response through eliminating excessive pro-inflammatory cytokines from the circulation thereby causing improvement in clinical status [13, 14].

Therapeutic plasma exchange (TPE) is a procedure of extracorporeal separation of plasma from blood and substituting plasma with a replacement colloid which aims to get rid of unwanted substances of blood. Evidence of using TPE in dengue infection is limited. It has been used in dengue associated acute liver failure, acute renal failure and dengue encephalopathy with fairly good outcome [15–17].

One of the major clinical challenges we faced here is gradually deteriorating GCS and persistent cholestasis with rising direct bilirubinemia of the patient.

Following TPE her GCS and overall clinical condition rapidly improved and was safely extubated. Her clinical improvement following TPE can be explained by neurotoxins and other cytokines being removed from the circulation.

The limitation we have identified in this case is that the patient was not screened for Hepatitis E infection due to the unavailability of facilities.

Dengue is mainly a disease in tropics. Due to the limitation of facilities, there is a knowledge gap and an inadequacy in evidence-based management in literature. Here we have reported a challenging case of ALF and intrahepatic cholestasis, a rare and a lethal complication of this common disease and our rough journey through the management of this patient with ultimate good outcome. This patient was managed successfully with supportive therapy, aided by cytosorb hemo-adsorption and therapeutic plasma exchange.

## Data Availability

Not applicable.

## Abbreviations

DHF: Dengue hemorrhagic fever
ALF: Acute liver failure
ARF: Acute renal failure
DIC: Disseminated intravascular coagulation
TPE: Therapeutic plasma exchange
NAC: N-Acetyl cysteine
GCS: Glasgow coma scale
ALP: Alkaline phosphate
GGT: Gamma glutamyl transferase
NCCT: Non-contrast computed tomography
CECT: Contrast enhanced computed tomography
CMV: Cytomegalovirus
EBV: Epstein bar virus
ANA: Anti nuclear antibody
AMA: Anti mitochondrial antibody
CRRT: Continuous renal replacement therapy
ROTEM: Rotational thromboelastometry
INR: International normalized ratio

## References

[1] Murray NEA, Quam MB, Wilder-Smith A (2013). Epidemiology of dengue: past, present and future prospects. Clin Epidemiol.

[2] Fernando S (2016). Patterns and causes of liver involvement in acute dengue infection. BMC Infect Dis.

[3] Yudhishdran J (2014). Dengue haemorrhagic fever presenting with cholestatic hepatitis: two case reports and a review of literature. BMC Res Notes.

[4] Thulaseedharan NK (2016). Review of dengue deaths: acute liver failure as a major cause of mortality. J Evid Based Med Healthc.

[5] Seneviratne SL, Malavige GN, De Silva HJ (2006). Pathogenesis of liver involvement during dengue viral infections. Trans R Soc Trop Med Hyg.

[6] Khongphatthanayothin A, Mahayosnond A, Poovorawan Y (2013). Possible cause of liver failure in patient with dengue shock syndrome. Emerg Infect Dis.

[7] de Macedo FC (2006). Histologic, viral, and molecular correlates of dengue fever infection of the liver using highly sensitive immunohistochemistry. Diagn Mol Pathol.

[8] Samanta J, Sharma V (2015). Dengue and its effects on liver. World J Clin Cases WJCC.

[9] Galvin Z (2015). Blood alanine aminotransferase levels> 1,000 IU/l–causes and outcomes. Clin Med.

[10] Kuo C-H (1992). Liver biochemical tests and dengue fever. Am J Trop Med Hyg.

[11] Kumar A et al. Prolonged neonatal cholestasis: a rare manifestation of dengue fever. Internet J Pediatr Neonatol. 2007; 9(1).

[12] Kumar S, Damera S (2020). Paediatric patient with dengue fever and associated multi-organ dysfunction syndrome (MODS) receiving hemoadsorption using Cytosorb® A case report on clinical experience. IJMDAT.

[13] Khan ZA (2016). A clinical experience of using extracorporeal cytokine adsorption device (Cytosorb®) in a case of dengue fever. J Evid Based Med Healthc.

[14] Wiegele M, Krenn CG (2015). Cytosorb™ in a Patient with Legionella pneumonia-associated rhabdomyolysis: a case report. ASAIO J.

[15] Prajong P (2020). Therapeutic plasma exchange and singlepass albumin dialysis in a 10-month-old girl with dengue shock syndrome in Thailand: a case report. Southeast Asian J Trop Med Public Health.

[16] Yadav KD, Mahay HS, Gupta R (2019). Therapeutic plasma exchange as a rescue therapy in three patients of severe dengue with hyperferritinemia and acute hepatic failure. Int J Surg Med.

[17] Thongthaisin A et al. Therapeutic plasma exchange as a treatment of a dengue encephalopathic patient. Case Rep. 2021;31(1).

